# Failure Mechanisms of Ba_0.5_Sr_0.5_Co_0.8_Fe_0.2_O_3−δ_ Membranes after Pilot Module Operation

**DOI:** 10.3390/membranes12111093

**Published:** 2022-11-03

**Authors:** Simone Herzog, Chao Liu, Nicolas Nauels, Anke Kaletsch, Christoph Broeckmann

**Affiliations:** 1 Institute for Materials Applications in Mechanical Engineering (IWM), RWTH Aachen University, Augustinerbach 4, 52062 Aachen, Germany; 2 Aachener Verfahrenstechnik (AVT), Mechanical Process Engineering, RWTH Aachen University, Forckenbeckstraße 51, 52074 Aachen, Germany

**Keywords:** Ba_0.5_Sr_0.5_Co_0.8_Fe_0.2_O_3−δ_ (BSCF), oxygen transport membrane (OTM), strength degradation, fracture probability, failure, creep relaxation, brittle ring test

## Abstract

The step from the testing of oxygen transport membranes on a lab scale to long-term operation on a large scale is a challenge. In a previous study, membrane failure was observed at defined positions of one end of the cooled tubular Ba_0.5_Sr_0.5_Co_0.8_Fe_0.2_O_3−δ_ membranes after an emergency shutdown. To understand the failure mechanisms, strength degradation and transient stress distribution were investigated by brittle-ring tests and finite element simulations, respectively. A 15% decrease in the characteristic strength of 162 MPa was proven after aging at 850 °C and was attributed to grain coarsening. The reduction in characteristic strength after thermal shock ranged from 5 to 90% depending on the cooling rates, and from 5 to 40% after the first and 20th soft thermal cycling. Simulations indicated the chemical strains induced by a 10-bar feed air and 50 mbar permeate pressure, which caused tensile stresses of up to 70 MPa at the outer surface. These stresses relaxed to 43 MPa by creep within a 1000 h operation. A remaining local stress maximum seemed to be responsible for the fracture. It evolved near the experimentally observed fracture position during a 1000 h permeation and exceeded the temperature and time-dependent strength. The maximum stress was formed by a chemical strain at temperatures above 500 °C but effective creep relaxation needed temperatures above 750 °C.

## 1. Introduction

According to the International Energy Agency, the cornerstones of decarbonization lie primarily in Increasing energy efficiency and CO_2_ capture, utilization, and storage (CCUS) [1]. A key technology for CCUS is the oxyfuel process for combustion under oxygen-enriched air or in pure oxygen. With the law to reduce and end coal-fired power generation that was passed in 2020, German coal-fired power plants will be decommissioned by 2038 at the latest [2]. The results of the two CO_2_ emission-free oxyfuel demonstration power plants “Schwarze Pumpe” (Germany, 2008–2014) and “Boundary Dam 3” (Canada, 2014-present) will therefore no longer be implemented in Germany. However, oxyfuel technology has also been tested for combined gas and steam power plants on a small scale [3]. Its implementation ultimately depends on the additional cost per ton of avoided CO_2_ emissions (50–100 $/t CO_2_ [4]) in relation to the price of CO_2_ allowances valid in the EU. If CO_2_ prices continue to rise as expected, CO_2_ capture by the oxyfuel process could become economically viable from 2030 onwards [5]. In addition to companies in the energy sector, raw material processing companies are also closely following the development of CO_2_ prices and costs through CO_2_ avoidance. Not only the iron and steel [4,6] but also the cement and glass industries [7], can achieve CO_2_ neutrality by oxyfuel combustion. Therefore, the oxygen demand is steadily increasing [8]. Besides these large potential consumers, there is also the need for a decentralized oxygen supply for medical applications [9], chemical industries, or wastewater treatment plants [10]. 

Depending on the required quantity, purity, and storage capacity, various processes are available for separating oxygen from the air. Conventional and established processes include cryogenic air separation and pressure swing adsorption. Oxygen can also be separated from the air, using ceramic membranes (oxygen transport membrane—OTM). At high temperatures (>750 °C) and different oxygen partial pressures on both sides of the membrane, the permeation of oxygen ions through gas-tight ceramics occurs. If a vacuum is set on one side of the membrane, while the oxygen donor (e.g., air, H_2_O(g)) flows on the other side (the feed side), pure oxygen can be obtained. This process variant is suitable for oxyfuel combustion and has been investigated on a laboratory scale with various membrane materials and geometries. Process simulations demonstrate cost savings by using OTM modules instead of cryogenic air separation, provided that they are advantageously and thermally integrated into the production or power plant periphery [3,7]. 

In OTM technology, there is already proof of operation for two major industrial O_2_ production plants and two research demonstrators. The progress made by Praxair (now Linde) and Saint-Gobain should be mentioned [11], who announced a four-end module with 300 tubular membranes and 1 TOPD (tons of oxygen per day) in 2014 [12]. Apart from that, Air Products and Ceramatec were already able to produce 100 TOPD with all-ceramic planar cell stacks made of La_1−x_Ca_x_FeO_3−δ_ in a three-end operation, and a 2000 TOPD system was announced in 2020 [13,14,15]. Meanwhile, tubular concepts focusing on the production of pure oxygen were presented by Fraunhofer IKTS [16] and the working group of TAN [17] with a hollow fiber demonstrator. Both modules operate without compressed air in the three-end mode. The hollow fiber module was able to achieve only 1/20 of its theoretical oxygen production of 0.05 TOPD but did achieve over 99% purity for about 1000 h. Various demonstrators at IKTS produced a maximum of 0.05 TOPD with operating times of up to 9000 h. A new demonstrator with a lower specific energy consumption per Nm^3^ O_2_ than pressure swing adsorption plants and theoretical production of 0.33 TOPD is patented [18] and is currently under development. 

Our previous publication reported on the test operation of a third research membrane module, the so-called Oxycoal-AC pilot module, with a theoretical membrane area of 14 m^2^ and a theoretical oxygen production of 0.6 TPDO [19]. The focus was on the design of the membrane module, flow and temperature distributions, and permeation rates in short- and long-term tests. A maximum oxygen purity of 96% and a maximum oxygen flux of 2.8 mL cm^−2^ min^−1^ were achieved, but an operating time of more than 1800 h could not be reached due to membrane failure. The membrane failures, which always showed the same typical fracture pattern, occurred either separately during the permeation operation or as mass failures after the emergency shutdown due to malfunctions of the control unit. 

In the case of the above-mentioned demonstrator systems as well as OTM laboratory systems, the reasons for membrane failure have only rarely been cited to date. Due to the unpopular publication topic of “membrane failure” there has been little insightful research addressing root-cause remedies. Hu’s recently published pilot test with seven asymmetric tubular zirconia doped Ba_0.5_Sr_0.5_Co_0.8_Fe_0.2_O_3−δ_ (BSCF) membranes in a demonstrator module that was terminated after 175 h [20]. During the experiment, unintended thermocycling due to a control malfunction occurred, but all the membranes survived. A hidden indication of membrane damage due to thermocycling is given in [14]. The more “events” (presumably failures) that occurred during the long-term testing of La_1−x_Ca_x_FeO_3−δ_ membranes, the lower the measured oxygen purity. Using a La_0.6_Sr_0.4_Co_0.2_Fe_0.8_O_3−δ_ plate as an example, Adler demonstrated how abrupt changes in the oxygen partial pressure could also lead to cracking as a result of chemical expansion (also known as stoichiometric expansion), analogous to the well-known thermal shock [21]. Rutkowski et al. reported severe leakage in BSCF membrane tubes after the module operation [22]. A scanning electron microscope showed elongated pores where cracks were initiated and had grown sub-critically. The cause was identified as control problems of the vacuum pump, resulting in high chemical strains and local stress concentrations for a short time. Several working groups were concerned with stress prediction, taking thermo-chemical strain into account [23,24,25,26]. The highly deformed planar LCF cell stack of Air Products after about 15,000 h of operation showed that creep deformation is also relevant for OTM modules [15]. Especially for asymmetric membranes with different creep rates of support and membrane layers, creep deformation is assumed to be the cause of delamination and the loss of gas tightness [24,27,28].

The scope of this work is to clarify the causes of membrane ruptures in the Oxycoal-AC pilot module. On one side, strength investigations that take into account the influence of the damage during the module operation and emergency shutdown are necessary. On the other side, a finite element simulation considering pressures, creep deformation, and chemical expansion is required to obtain a transient stress distribution. This approach allows the calculation of the fracture probability and identification of necessary design or operation adaptions to potentially contribute to reliable module constructions in the future.

## 2. Materials and Methods

### 2.1. Membrane Geometry

One side of the closed membranes tubes (inner diameter d_i_ = 13.9 mm, outer diameter *d_o_* = 15.5 mm, length *l* = 500 mm) was fabricated by the cold isostatic pressing of Ba_0.5_Sr_0.5_Co_0.8_Fe_0.2_O_3−δ_ granules (mean grain size 3 µm, mean granule size 125 µm) onto a steel core and were subsequently sintered between two Al_2_O_3_ tubes. Details on the process and material, as well as the joining of metal sleeves using an epoxy resin, are given in [19]. 

**Figure 1:**
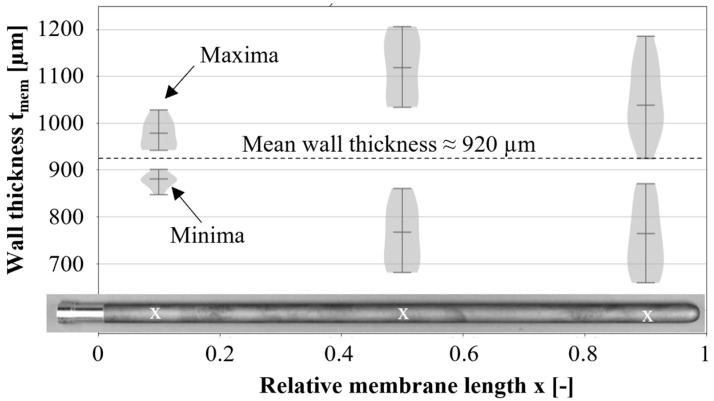
Non-destructively measured distribution of minimum and maximum wall thickness of membrane tubes. X indicates the measuring position.

In order to reproducibly produce gas-tight membranes with a homogeneous wall thickness of about 0.8–1 mm, the press molds and types of vibration were varied during the filling of the granulate. With non-destructive wall thickness measurements (Minitest 7400FH, Electrophysics), 10 membrane tubes each were measured at three positions at a distance of 50, 250, and 450 mm from the open membrane end. In the following report, we will give positional data normalized to the total membrane length of 500 mm, where the relative membrane position x = 0 describes the joint at the open end and x = 1 at the closed membrane end. The minima and maxima at one position were used to determine the wall thickness distribution. An example of the characteristic wall thickness distribution is shown in Figure 1, where the latest developed pressing mold with a vertical vibration of the granules during filling was used. The largest inhomogeneities of the wall thickness Δt_mem_ occurred between x = 0.5 and 1. At the joining zone (x = 0), the wall thickness was relatively homogenous with ∆t_mem_ < 200 µm. The mean wall thickness amounted to 920 µm. When the vibration was horizontal, the scattering increased. Pressing molds used in the past resulted in thinner membrane tubes with an average wall thickness of 820 µm, and the largest inhomogeneity ∆t_mem_ = 500 µm was at the open membrane end [29]. 

In addition to inhomogeneous wall thickness (also referred to as eccentricity), longitudinal curvature and ovality can also occur in the tubular membranes. These imperfections are reported in [30] as characteristic imperfections for the extrusion process. The membranes in our study did not show any measurable curvature or ovality.

### 2.2. Module Operation and Membrane Failure

**Figure 2:**
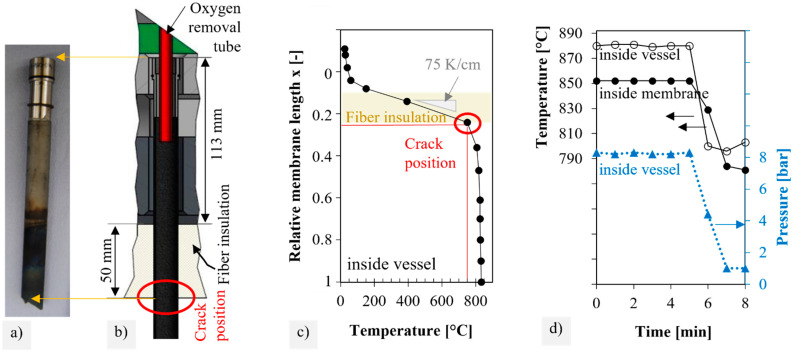
Typical fracture position (**a**) as photograph or (**b**) as sketch visualizing the mounted membrane in the module flange. (**c**) Axial temperature gradient inside membrane during permeation operation; (**d**) temperatures and pressures during emergency shutdown (t = 5 min).

As described in [19], the steel sleeves are pressed into the water-cooled flange and sealed by O-rings. To attenuate the gradient from the 850 °C hot vessels to the water-cooled flange, fiber insulation was mounted below the flange. All membranes failed at a similar position, just below the exit from the fiber insulation of the flange, as shown in Figure 2a,b). At this position of 12.5 ± 1 cm distance from the sealing position (x = 0.25), the membranes showed a gray-bluish discoloration. 

Analyzing the axial temperature gradient along the membrane length, Figure 2c indicates prevailing static temperatures of about 750 °C at the typical fracture position. Figure 2d presents exemplarily the pressure and temperature drop that was measured during an emergency shutdown. The temperature was moderately reduced by 80 °C within 2 min, while the feed pressure dropped completely to an ambient pressure during this time.

### 2.3. Brittle Ring Tests

The brittle ring test was selected to investigate the strength of the BSCF after pre-treatment, as this allowed the direct separation of the specimens from the membrane tubes so that the same defect distributions were present in the volume of the specimens as in the membrane tubes of the pilot module. 

Sintered and partially pre-treated membrane tubes were prepared into circular rings with a diamond cutting blade under isopropanol cooling. The segment edges were then ground with a diamond tool disc until no more chipping was visible. In an improved preparation variant, the edges were subsequently wet-polished with abrasive papers with 80, 150, and 300 grit so that optically shiny edge surfaces were achieved.

A total of 178 brittle ring specimens with an outer diameter of *d_o_* = 15.5 mm, a wall thickness of *t_mem_* ~920 µm, and a length of *l* = 10 mm were produced in this way. In some cases, the wall thickness of the membrane tubes was measured non-destructively (see Section 2.1) at the position of sampling before cutting. The experimental plan is summarized in Table 1. 

Since two different edge qualities, namely “Polished” and “Ground” were applied, two different Reference Series “1” and “2” were also tested to calculate the strength degradation. To allow the application of Weibull statistics, the intended number of specimens per series was 20. The following pre-treatment procedure was applied to the series A–D before strength testing:

- Series A: Pre-treatment was performed on the finished brittle ring specimens. For each test series A1–A4, a specific cooling was performed after homogenization at 850 °C for 30 min in a chamber furnace: furnace opening (Series A1), removal out of the furnace (Series A2), removal out of the furnace and fan cooling (Series A3), and water quenching (Series A4). Temperatures were recorded using three calibrated type K thermocouples wrapped/pressed to the membrane segments. Measured and fitted cooling profiles are given in Appendix A.
- Series B: The brittle ring specimens were prepared from still-intact membrane tubes after long-term permeation below the discolored area (position x > 0.35). The operating time of the membranes in the module varied from 1000 h to 1800 h [19].
- Series C: Specimens were cut from the discolored areas (position 0.2 < x < 0.3 mm) near the typical fracture origin of the membranes after the termination of long-term tests in [19] to investigate if a chemical reaction reduced the strength. During the specimen preparation, a different form of edge polishing compared to that in the A, B, and D series was carried out. Repeating the experiment was not possible due to the limited areas of blue discolored membranes. Therefore, reference series 2 was made from sintered membrane tubes with an identical edge quality and was tested.
- Series D: Strength degradation by thermal cycling.
  In a laboratory module, membranes were subjected to a total of 20 heating and cooling cycles between 100 °C and 850 °C with a heating rate of 150 K/h and natural cooling to 100 °C within 7.5 h (see Appendix B). No membrane failure occurred during the cycling tests. However, an operator error applied a slight internal overpressure of 50 mbar at the end of the test causing two membranes to fail at the typical fracture position. To check the residual strength after thermocycling and short-term internal pressure, a total of eight circular ring specimens were separated from the two remaining intact membrane tubes.

Prepared brittle ring specimens were positioned with their lateral surfaces between two plane-parallel plates so that a linear contact resulted and was loaded until fracture. Fracture tests were performed with a universal testing machine (model 8562, Instron) with a 10 kN load cell and a testing speed of 0.2 mm/s. The machine stopped at a force drop of 80%. The test time taken was less than 15 s, as required in DIN EN ISO 843-1 to suppress the effects of subcritical crack growth. With the fracture force F the tangential stress σ can be calculated as [31]:

(1) $$\sigma =\frac{F\left(3d_{o}-t_{mem}\right)}{l\pi t_{mem}{}^{2}}.$$

The measurement uncertainty of the flexural strength of $u_{\sigma }$ was calculated by the Gaussian error propagation for independent error-prone variables of Equation (1)

(2) $$u_{\sigma }=\sqrt{{\left(\frac{3d_{o}-t_{mem}}{l\pi t_{mem}{}^{2}}u_{F}\right)}^{2}+{\left(\frac{3F}{l\pi t_{mem}{}^{2}}u_{do}\right)}^{2}+{\left(\frac{F\left(t_{mem}-3d_{o}\right)}{l^{2}\pi t_{mem}{}^{2}}u_{l}\right)}^{2}+{\left(\frac{F\left(t_{mem}-6d_{o}\right)}{l\pi t_{mem}{}^{3}}u_{t}\right)}^{2}}.$$

Hereby, the measurement inaccuracy of the force $u_{F}$ was 0.1 N, the inaccuracy of the diameter $u_{do}$ was estimated to be 0.1 mm, and the inaccuracy of the length $u_{l}$ was 0.005 mm. The error on the wall thickness $u_{t}$ was calculated as the mean value between the maximum wall thickness $t_{max}$ and minimum wall thickness $t_{min}$ of each specimen determined in the reference 1 series separately. 

Closed numerical equations existed for the calculation of the effective volume [32]. Nevertheless, these are based on the assumption of a plane stress state and are no longer valid for specimens with a ratio of *l/*$t_{mem}$ > 6. A simulation of the stresses using the finite element (FE) method is therefore proposed in [33] and also used in this work (see Section 2.6). 

### 2.4. Creep Rupture Tests Using Tubular Membranes

**Figure 3:**
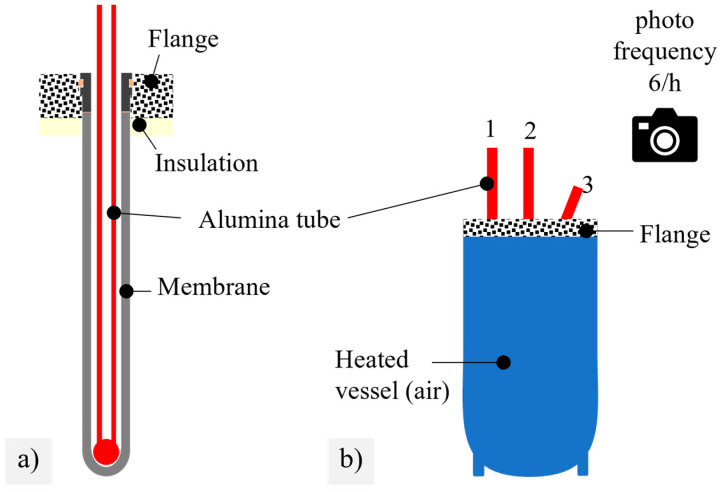
(**a**) Section through membrane loaded by alumina tube; (**b**) experimental view with failure of membrane 3. Drawings are not to scale.

The lifetime of BSCF membranes under static axial tensile stresses was determined by a simple photographic documentation method. For this purpose, 15 membrane tubes were glued to metallic sleeves and fixed in the water-cooled flange of a small laboratory module and subjected to stresses in the range of 3.8–6.3 kPa by inserting Al_2_O_3_ tubes with rounded ends and a defined weight, see Figure 3a. Note that all the membranes passed a quality assurance described in [19] comprising leak-tightness under 2 bar inner and 26 bar outer air pressure. Photographs were taken at intervals of 10 min. During heating with 100 K/h, no membrane failures occurred. The temperature was kept isothermal at 850 °C until the rupture of the last membrane tube. A membrane failure was identified by a shifted position of the Al_2_O_3_ tube between two successive images, as illustrated for membrane 3 in Figure 3b. The lifetime was calculated from the image number.

### 2.5. Surface and Microstructure Characterization 

After module operation, membrane parts were taken near the fracture position and near the closed end. After cleaning under compressed air, images, as well as energy-dispersive spectrometry (EDS) measurements, were taken with the scanning electron microscope (SEM) (Jeol JSM 6400). To assess the uncertainty of the quantitative EDS measurements on the curved and rough membrane surface, the wavelength-dispersive spectrometry (WDS (Cameca Microprobe) of polished, sintered BSCFs were used. The characteristic X-ray intensities obtained in EDS were corrected by two methods. A “ZAF”-correction considering the effects of the atomic number (Z), absorption (A), and fluorescence (F) was performed first, followed by a correction of the measured carbon content, which originated from the oil of the vacuum pump. X-ray diffraction (XRD) analyses were performed for the phase analysis using Cu K_α_ radiation (Seifert XRD 3003 PTS).

Cross sections were made to compare porosity between the sintered and module-aged BSCF. Porosity was determined on randomly positioned BSE images in ImageJ2 software [31]. EDS line scans were taken on the same samples to identify the differences in chemical composition along the membrane wall. Grain size analysis was performed by image analysis using BSE images of thermally etched sections (1000 °C without hold time) with AnalySIS^®^ software. For grain boundary detection, the grain boundaries were manually traced in black. In the binarized image, the equivalent diameters were determined from the detected grain areas and assigned to equidistant bins of a 5 µm width. The class boundaries were multiplied by a factor of 1.273 to account for the transfer from 2D image to 3D volume [32,33]. Similarly, the pore size distribution was determined in ImageJ2.

### 2.6. Finite Element Simulation and Fracture Probability 

**Figure 4:**
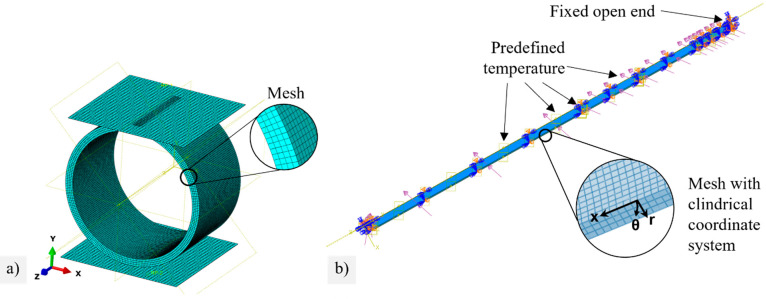
FE model of (**a**) the brittle ring test with structured mesh, (**b**) a membrane tube with boundary conditions and structured mesh in the pilot module.

To simulate the stress distribution and calculate the effective volume of the circular ring tests, a model with a linear contact to the support and load surface was set up in Abaqus and meshed with linear brick elements with a reduced integration point C3D8R, see Figure 4a. Linear-elastic material behaviors with characteristic values given in Annex 4 for the BSCF in the air were assumed. 

For the simulation of pressures in chemically induced stresses and cooling due to emergency shutdowns, ¼ of the membrane tubes were created with symmetry constraints in the x-y and y-z planes, see Figure 4b. The open end of the membrane tube was fixed. Meshing was performed with C3D8T elements to represent coupled displacements, temperatures, and pressures. Based on this initial state (step 0), three further steps were simulated (see Table 2). Step 1 applied the static temperature field, as shown in Figure 2c, by defining analytical fields along the axial direction. The compressive and tensile loads caused by feed and permeate pressures were applied on the outer and inner surfaces, respectively. In addition, the chemical strain was considered by using subroutine USDFLD. Based on this stress state, the creep relaxation within the first 1000 h was calculated by subroutine CREEP in step 2. The emergency shutdown was simulated in step 3 by decreasing the compressive loads on the outer surface immediately and the temperatures by 10% within 120 s (similar to Figure 2d). The chemical strain did not decrease spontaneously but rather dropped linearly to 0 within the 120 s to match the kinetics of oxygen incorporation into the lattice. Further details, used material parameters, and the corresponding references are summarized in Appendix D. The subroutine code is available in [34].

To calculate the effective volume *V_eff_*, the equivalent stress was formed at each integration point *i* according to the normal stress hypothesis, where n describes the total number of integration points. With the Weibull modulus m, the maximum principal stress in the whole model *σ_I,max_* and the integration point volume *V_i_*, the effective volume assuming the principle of independent action (PIA) was obtained with:

(3) $$V_{eff}=\int_{i=1}^{n}{\left(\frac{\sigma_{I,i}+\sigma_{II,i}+\sigma_{III,i}}{\sigma_{I,max}}\right)}^{m}\cdot V_{i}di.$$

According to the normal stress hypothesis, Equation (4) simplifies to:

(4) $$V_{eff}=\int_{i=1}^{n}{\left(\frac{\sigma_{I,i}}{\sigma_{I,max}}\right)}^{m}\cdot V_{i}di.$$

The fracture probability $P_{f}$ was calculated using the Weibull modulus m and the normalized stress $\sigma_{0v}$ as follows:

(5) $$P_{f}=1-exp\left[-\frac{V_{eff}}{V_{0}}\cdot {\left(\frac{\sigma_{I,max}}{\sigma_{0v}}\right)}^{m}\right].$$

In the component design, data from fracture tests of a specimen geometry (index 1) are usually used to calculate the fracture probability of a real component geometry (index 2). For the same materials as well as defect distributions $\sigma_{0,v1}=\sigma_{0v,2}$ and $m_{1}=m_{2}$ applies. Assuming equal fracture probabilities $P_{f,1}=P_{f,2}$, we obtain the so-called size effect equation

(6) $$\sigma_{2}={\left(\frac{V_{eff,1}}{V_{eff,2}}\right)}^{1/m}\cdot \sigma_{1},$$

with the fracture stress of the specimen $\sigma_{1}$ and the component $\sigma_{2}$, respectively. Analogous to the size effect equation, failure due to subcritical crack growth can be specified at different times $t_{i}$ for the same failure probability

(7) $$t_{2}={\left(\frac{\sigma_{1}}{\sigma_{2}}\right)}^{n}\cdot t_{1}$$

where n is the stress exponent.

## 3. Results

### 3.1. Surface and Microstructure Modifications by Module Operation

**Figure 5:**
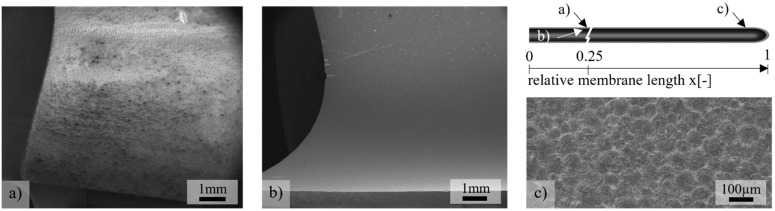
SE-images of a failed tubular membrane after 1800 h module operation, (**a**) blue discolored outer surface (fracture position), (**b**) corresponding inner surface, (**c**) outer surface exposed to 850 °C.

The outer, blue discolored area near the typical fracture position in Figure 5a appears rough and irregularly covered. XRD phase analysis did not show any additional peaks besides BSCF (see Appendix C). However, reliable diffraction measurements on rough and curved surfaces are a challenge. The corresponding inner side of the membrane in Figure 5b is so smooth that some pores are also visible. With higher magnification at the closed end of the membrane, a granular structure becomes visible, as shown in Figure 5c. This surface structure is present along the entire membrane length, presumably also below the coating at the fracture position.

The chemical composition of the inner membrane surface, measured with EDS, hardly deviated from the WDX reference measurement, see Table 3. Despite the curved surface and carbon contamination, the C-corrected EDS values were robust for comparative analysis. The blue discoloration at the fracture origin occurred together with the newly detected elements of aluminum, silicon, sulfur, and chromium, whereby the chromium content varied strongly.

**Figure 6:**
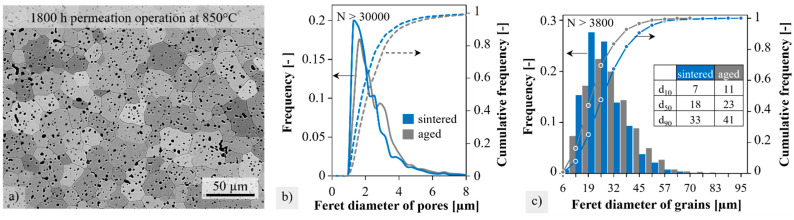
(**a**) Exemplary BSE image of BSCF after thermal etching, (**b**) pore sizes and (**c**) grain size distribution of BSCF in the sintered condition and after 1800 h aging at 850 °C in module permeation.

Comparing micrographs for the sintered BSCF with those after module operation revealed no obvious differences regarding the shape or the distribution of pores and grains. An exemplary SEM image of etched BSCF after 1800 h of module operation is shown in Figure 6a. Numerous, mostly spherical, and isolated pores occurred, which were predominantly intracrystalline. Grain boundaries are often curved as they are pinned by the few intercrystalline pores during the final sintering stage. Pore clusters rarely occur. Quantitative image analysis revealed minor differences between the sintered and module-aged membrane tubes. Image sections from the inside and outside of the membrane were equally included in the evaluation since no differences were detected here. In contrast to [35], we did not measure the gradients of A and B cations in EDS line scans due to kinetic demixing. The total porosity increased from 4.2 ± 0.3% to 5.8 ± 0.5%. This was accompanied by a slight coarsening of the pores, as seen in Figure 6b. The volume-corrected Feret diameter d_50_ increased from 1.8 to 2.1 µm, and d_90_ increased from 4.0 µm to 6.3 µm after aging in the pilot module. The differences are small but statistically robust due to the large number of pores (>30,000) and image sections (7) examined. 

The grain size analysis in Figure 6c also demonstrates the trend towards coarsening in the histogram by comparing the characteristic grain diameters d_10_, d_50,_ and d_90_ of the sintered and aged membranes. They were increased by 4, 5, and 8 µm, respectively. The operating temperature of the module (850 °C) was 78% of the sintering temperature (1100 °C). Thus, the observed grain coarsening was mainly due to “post-sintering”, where, typically, the large grains grow at the expense of the small grains.

### 3.2. Reference Strength

**Figure 7:**
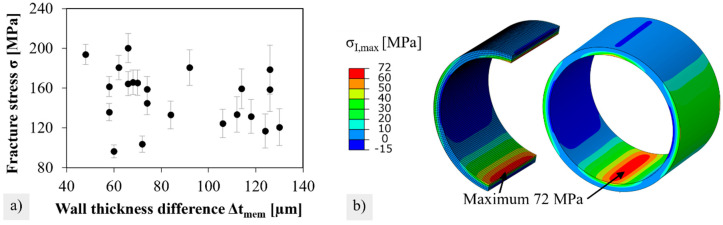
(**a**) Data cloud of fracture stress versus wall thickness difference for reference series 1. (**b**) Stress distribution in a brittle ring specimen.

The determined fracture stress in the dependence of each individual specimen’s wall thickness difference from reference series 1 is depicted in Figure 7a. The data points seem randomly distributed, and a clear correlation between fracture stress and wall thickness difference could not be found. Error bars were obtained from the Gaussian uncertainty propagation in Equation 2. In principle, the four individual uncertainty variables on the force $u_{F}$, the outer diameter $u_{\mathrm{do}}$, the length $u_{l,}$ and the wall thickness $u_{t}$ contribute to the total error of the stress. However, the error term with the error variable $u_{t}$ superimposes all the other influencing variables, so that $u_{\sigma }$ is almost exclusively determined by $u_{t}$.

The stress distribution in the brittle ring test is shown in Figure 7b. Note that maximum principal stresses at the upper and lower inner sides are about twice as much as those at the lateral outer sides. The volume elements positioned at the inner side contribute significantly to the failure of relevant effective volume.

Experimentally determined Weibull parameters, simulation-determined effective volumes, and the calculated normalized strength of both reference series are summarized in Table 4. Overall, the normalized stress and Weibull modulus of Reference 1 are in a similar range to those determined in the ball-on-ring test (179 MPa and 7.1, [36]) and ball-on-three-balls test (189 MPa and 8.8 [37]) where the BSCF had a porosity of approximately three percent. The reduced Weibull parameters in Reference 2 are not unexpected since edge defects cause stress concentrations. All fracture tests in this work were conducted at room temperature. However, from [38] we know that there is a decrease in the Weibull modulus by 16% and the normalized strength by 39% when increasing the testing temperature to 850 °C. As a rough estimation for the fracture probability calculation, these 850 °C-scale factors were applied to Reference 1 to form Reference 1 *.

### 3.3. Strength Degradation Obtained by Pre-Treatment

**Figure 8:**
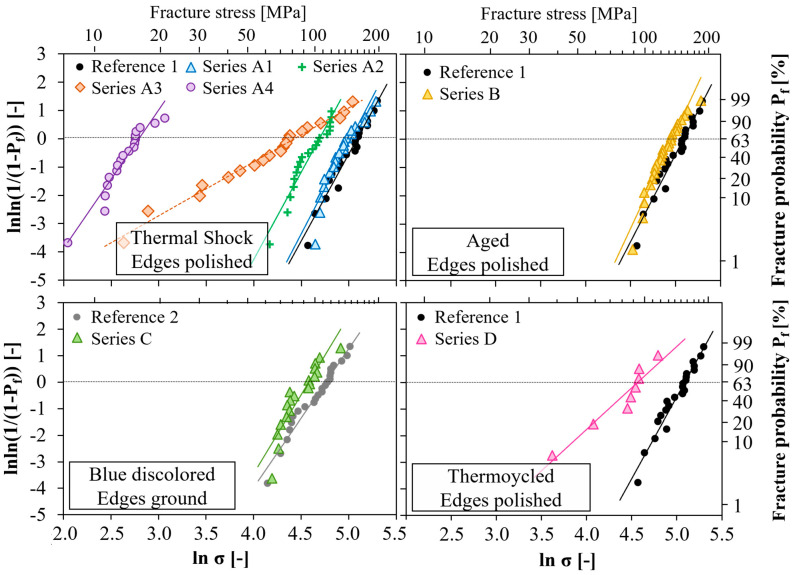
Weibull plots of brittle ring tests after pre-treatment.

Figure 8 summarizes the Weibull plots determined for the test series A–D after pre-treatment and the corresponding reference series without pre-treatment.

The Weibull parameters, the effective volume, and the resulting normalization stress of the different sample series are given in Table 5. Due to the very small Weibull modulus in series A3 and D, the effective volume became large. In fact, such a low Weibull modulus indicates that the component is quite unreliable, and consequently, the corresponding normalized strength is no longer technologically applicable.

The degradation of characteristic strength with increasing cooling rates during the thermal shock treatment from series A1, A2, and A3 to series A4 is evident (see Figure 8a). It was not expected that such severe strength reductions could occur in such thin-walled specimens due to thermal shock. The distribution of the critical defect type obviously changed from test series 3 onward since the Weibull modulus showed a sharp jump from about six to two. It has to be taken into account for series A3 and A4 that thermal shock already caused fractures in two and three specimens, respectively. For reasons of comparability, the premature failures were excluded from the Weibull evaluation but were included as a superscript in the Weibull parameters in Table 5. Micrographs were taken to evaluate the microstructural damage due to thermal shock. in contrast to the reference specimen, microcracks formed after thermal shock (Appendix A). As expected, most of these cracks ran in a radial direction. Since the outer surface cooled faster than the solid body, the temperature and consequently stress gradient were greatest in the radial direction. The crack length varied between 20 and 200 μm. However, a quantitative correlation between the crack density and cooling rate could not be derived.

The described microstructural changes during the module operation in the hot zone at 850 °C, as described in Section 3.1, affects the strength negligibly, as seen in Figure 8b. The same applies to specimens with discolored membrane sections (see Figure 8c), which were exposed to intermediate temperatures and were subsequently prepared. Note that the strength degradation of Series C has to be assessed with the reference Series 2 since the edges were not polished, only the ground.

In Figure 8d, the results after thermocycling are plotted together with reference series 1 results. Since the number of specimens is very small (*n* = 8), the usual data evaluation, according to Weibull, is subject to great uncertainty. Nevertheless, the trends of a strength decrease (162 to 96 MPa) and an increase in the scatter (six to three) are undoubtedly recognizable by the decreased slopes and shifts to the left.

### 3.4. Tubular Membranes under Static Axial Tensile Stress

**Figure 9:**
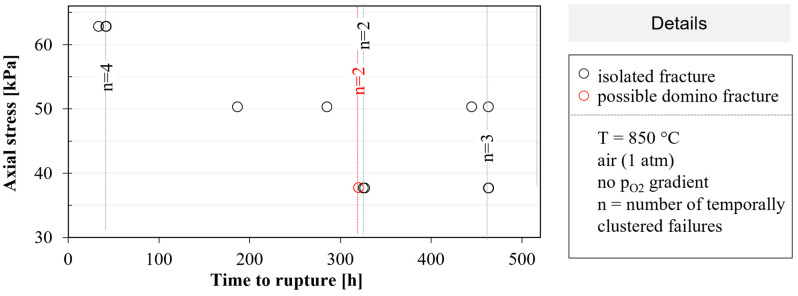
Lifetime of membranes under axial tensile stress; the number n of temporally clustered failures is indicated and marked by dashed lines.

Based on photo documentation, the time to rupture was obtained for each membrane. The heating elongation of several millimeters was observed qualitatively, as expected, from the thermal expansion. Since no rupture occurred during heating, the results are shown in Figure 9 from the beginning of the isothermal phase at 850 °C (t = 0 h). Membrane tubes under higher tensile stresses failed earlier in the trend without remarkable creep elongation. Out of a total of 15 membranes, nine membranes failed one after the other in 10-min intervals (corresponding to the photo frequency). These failures with the associated number of samples n are marked by dashed lines in Figure 9. After 320 h, two membranes, which are marked in red, failed possibly at the same time (two failures in one photo). Except for one of these two membrane cases, a domino effect might have occurred (1 out of 15). At least 93% (1–1/15) of a membrane tubes failed due to static fatigue loading. 

### 3.5. Stress Distribution and Predicted Fractuire Probability during Module Operation

**Figure 10:**
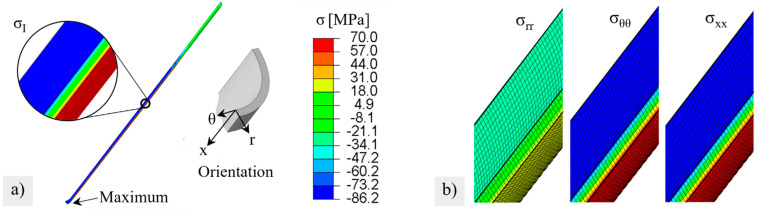
Stress distribution after virtual heating and pressurizing: (**a**) maximum principal stress (**b**) stress components consisting of radial $\sigma_{rr}$, tangential $\sigma_{\theta \theta }$ and axial stresses $\sigma_{xx}$ at the relative membrane length x = 0.5. The color legend is valid for (**a**,**b**).

The simulated stresses, after heating and loading under 10-bar external pressure and −50 mbar internal pressure (at the end of Step 1 in Table 2), are shown in Figure 10a. A very high stresses of up to 70 MPa was induced by chemical expansions that occurred on the outside, which counteracted the external compressive stress. The high normal stresses acted in the tangential and axial directions in the hot membrane zone, see Figure 10b. The maximum stress was reached at the closed end of the membrane.

**Figure 11:**
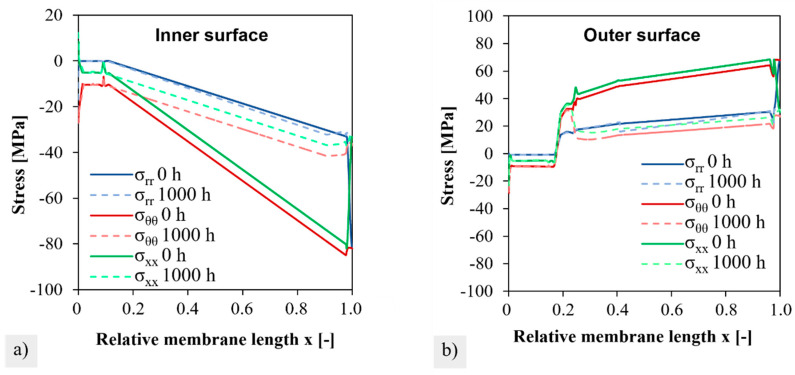
Change in stress components during 1000 h virtual module operation with creep relaxation. $\sigma_{rr}$ ist the radial, $\sigma_{\theta \theta }$ the tangential, and $\sigma_{xx}$ the axial stress.

The distribution of the stress components from the fixed position to the closed end of the membrane on the inside and outside of the membrane is shown in Figure 11a,b), respectively, at the beginning (t = 0 h) and the end of creep (t = 1000 h). Due to the fixation (x = 0) and the rounding of the closed membrane end (x = 1), deviations from the general smooth stress profile occurred. However, these positions were not relevant to failure during module operation. During creep, the stress components on the inside were equalized. On the outside, the tangential $\sigma_{\theta \theta }$ and axial stresses $\sigma_{xx}$ remained unchanged up to position x = 0.21 (≈11 cm) and decreased thereafter. This led to a local maximum after 1000 h of creep. For very long times of >1000 h, it could be predicted that the stresses on the inside and outside would equalize to fully relax the chemical strains, as described in [30]. In the transition region and in the cold zone, the creep rates are obviously not sufficient for relaxation.

**Figure 12:**
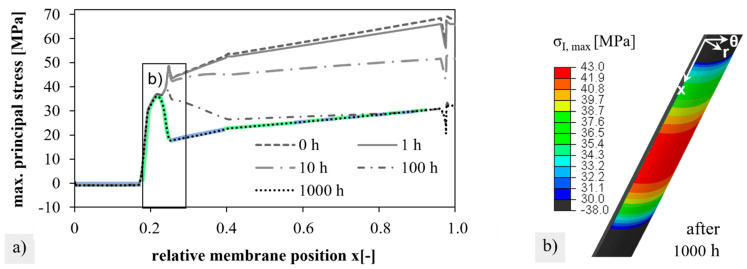
Maximum principal stress on the membrane outside after different creep times; (**a**) plotted versus the relative membrane length; radial (blue) and axial (green) stress components are marked. (**b**) Distribution around the stress maximum.

The resulting local stress maximum and its time-dependent formation are also visible in the course of the first principal stresses on the outside in Figure 12a. After 1000 h, the first principal stress was followed by the course of the radial stress (blue) and the axial stress (green). From the time course of the stress components (as compared in Figure 11b), it can be estimated that even with creep times above 1000 h, the local stress maximum is maintained, whereas stress levels of areas located further down (x > 0.25) will still drop slightly.

**Figure 13:**
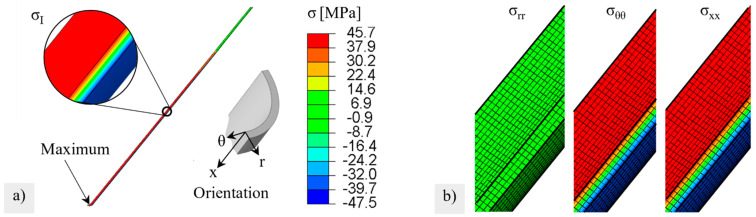
Stress distribution after pressure relieve and 10% cooling within 120 s: (**a**) maximum principal stress (**b**) stress components at the relative membrane length x = 0.5. The color legend is valid for (**a**,**b**).

In simulation step 3, the pressures were linearly removed within 2 min. Meanwhile, the temperature of the inner and outer surfaces was assumed to decrease exponentially by 10% during cooling (see Appendix D). The results are given in Figure 13a). Overall, cooling during the emergency shutdown with the assumed parameters within the wall thickness did not lead to higher temperatures and stress gradients. The maximum temperature difference between the surfaces and the membrane interiors was about 50 K resulting in a stress difference of 2 MPa. The pressure dropped, and the equalization of the oxygen partial pressure relieved chemically induced strains/stresses. Since the external pressure was absent as a counterforce, the membrane tube expanded, causing internal axial and tangential stresses (Figure 13b). These increased linearly from the position x = 0.1 with *σ_θθ_* = 2 MPa and *σ_xx_* = 0 MPa, respectively, to 47 MPa at the end of the membrane.

In the simulation of the membrane tube, the separate steps (1)—start, (2)—permeation, and (3)—emergency shutdown were analyzed to calculate the effective volume with Equation (4). In addition, two sets of Weibull parameters and two different failure hypotheses (the normal stress hypothesis (NS) and the principle of independent action (PIA)) were varied in the fracture probability calculation. Table 6 presents the results.

Regardless of which assumptions were made for the Weibull parameters or which failure hypotheses were selected, a fracture probability of 100% was obtained for a single membrane tube after heating and pressurizing (step 1). After creep relaxation within 1000 h of permeation operation (step 2), the maximum principal stress and thus effective volume decrease, resulting in low failure probabilities of 6–8% with Weibull parameters from [38]. Emergency shutdown (step 3), while assumed to be more critical than a static permeation operation, was calculated almost as relevant as the failure of a module start-up (step 1) with fracture probabilities of nearly or exactly 100%. However, no membrane ruptures were observed during start-up under the applied feed pressure [19]. Overall, a large discrepancy between the predicted failure probability and experimental observation remains to be noted. A comparison between the normal stress and PIA hypothesis showed larger effective volumes by a factor of 1.3–1.9 when all three stress components of the PIA hypothesis were considered. This can be justified by the previously described stress condition with equally high tangential and radial stresses. In the NS hypothesis, one of these stress components is not considered.

## 4. Discussion

### 4.1. Limitations of the Brittle Ring Test

In the simulation, a homogeneous wall thickness of the membrane tubes of 920 µm was assumed. By means of non-destructive measuring techniques, however, inhomogeneities were detected, which had a global minimum of 650 µm at the end of the membrane and local minimum values of about 750 µm at the typical fracture position. In the Gaussian error propagation (Equation (2)), the wall thickness uncertainty $u_{t}$ had a significant influence on the uncertainty of the calculated flexural strength. For example, the deviation of the wall thickness from the mean wall thickness $\bar{s}$ = 920 µm described above caused variations in the applied stresses from 86 MPa (t = 920 µm) to 114 MPa (t_min_ = 800 µm) at the typical fracture position or 129 MPa (*t_min_* = 750 µm) at the membrane end (assumptions: *l* = 10 mm, *F* = 50 N, *d_o_* = 15.5 mm, t from Figure 1). However, positioning the brittle ring specimens relative to the thinnest wall thickness can determine whether there is an underestimation of the actual maximum stress σ_max_.

**Figure 14:**
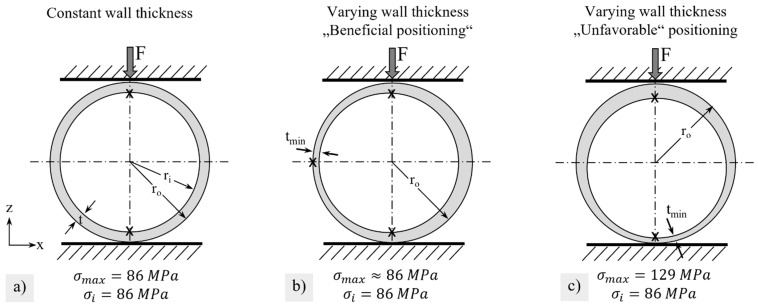
Schematic representation of how the positioning of brittle ring specimens with inhomogeneous wall thickness influences the stress σ_max_ at failure and the fracture stress σ_i_ determined in the test. Assumptions: l = 10 mm, F = 50 N, d_o_ = 15.5 mm, t_min_ = 750 µm. (**a**) constant wall thickness, (**b**) inhomogeneous wall thickness with beneficial positioning, and (**c**) inhomogeneous wall thickness with unfavorable positioning.

If the minimum wall thickness was located at the side (see Figure 14b), the average wall thickness used in the stress calculation was located at the highly loaded position. Possibly, the outer sides would also be relevant for failure in this case. With homogeneous wall thicknesses (Figure 14a), however, a brittle ring will always fail at the inner sides positioned at the top and bottom since the FE simulations in Figure 7b determined almost twice as high stresses here as at the lateral outer sides. In the case of ‘unfavorable’ positioning (Figure 14c), high stresses σ_max_ could occur at the locally thinnest wall thickness, but these are underestimated when calculating the fracture stress of the circular ring σ_i_ by means of Equation (1) with the average wall thickness of the specimen.

The theoretically derived influence of an inhomogeneous wall thickness on the fracture stress of circular rings could not be demonstrated with the data from reference series 1 in Figure 7a. The reason for this was probably the pre-selection, which excluded circular rings with an Δt > 150 µm, and thus resulted in small stress differences between σ_max_ and σ_i_. The positioning effects were ultimately super-positioned by the inherent material scatter in strength due to defect distributions. Thus, the technological Weibull modulus from the brittle ring test is not a pure material parameter anymore in the case of inhomogeneous wall thicknesses. This could also explain why the Weibull modulus of reference series 1 with m = 6.2 is slightly reduced in comparison to values obtained by Zwick (m = 7.1 in the Ball-on-ring test [36]) or Herzog (m = 8.8 in the Ball-on-3-balls test [37]).

Series C comprises blue discolored membrane rings which were exposed to intermediate temperatures in the membrane module. However, in contrast to series A, B, and D, the edges were not polished but only grounded, so that Reference 2 of the sintered membrane tubes with ground edges was used for comparison purposes. Figure 8c) shows a 16% decrease in the characteristic strength (117 to 98 MPa) with an insignificant increase in the Weibull modulus. The changes are similar to those already observed between series B and Reference 1 (Figure 8b). The lower stress level of series C was due to edge defects that were not removed by polishing. Coming back to the FE simulations, which attested twice as high stresses at the top/down inner side than the lateral outer sides, we concluded that the brittle ring test was not a suitable method but proved a possible strength degradation caused by reaction layers at the outer surface.

### 4.2. Correlations between Microstructure and Strength

In previous works, normalized stress after 1500 h of isothermal aging at 850 °C was decreased by 22% from 189 MPa [37] to 148 MPa [39] for the BSCF tested in the ball-on-three-balls test. This is in good agreement with series B, where the normalization stress was decreased by 16% from 218 to 183 MPa after 1800 h of aging in the pilot module (Reference 1 vs. series B). If the brittle ring specimens from the blue colorized area are considered (Reference 2 vs. series C), the decrease in normalization stress amounts to 22%. The strength degradation of series B and C is predominantly attributed to the general microstructure condition of the aged membranes.

The comparative grain size determination was proofed grain coarsening after the module operation. The determined values for grain coarsening after 1800 h of module operation tended to be comparable with values from Kaletsch [39]. During artificial aging at 850 °C in the air for 1500 h, changes in the d_10_, d_50_, and d_90_ of 50, 38, and 31%, respectively, were measured, although the overall grain size was significantly higher in Kaletsch’s study. In addition, the global porosity increased from 4.2% to 5.8%, with a simultaneous increase in the pore diameter d90 from 4.0 µm to 6.3 µm. Pore coarsening due to Ostwald ripening was not unexpected. The qualitative observation of grain coarsening and pore coalescence in the LSCF and LSC membranes by Lein et al. [40] confirms our observations. However, regarding the total porosity, we expected the opposite effect since intergranular porosity can be reduced by mass transport as a result of post-sintering. The increase in the total porosity may thus have two different roots: firstly, in the pore size analysis, only pores above the threshold of 1 µm diameter were detected, as the magnification was chosen to allow a representative area to be examined. It cannot be ruled out that in the sintered condition, many small pores could have existed which were below the detection limit and only became measurable as a result of pore coarsening. Secondly, the increase in the total porosity might be caused by the diffusional vacancy transport itself. It was not observed in the artificial aging of Kaletsch under air [39]. 

Chemical modifications of the membrane after permeation are more frequently and comprehensively studied in the literature. The coexisting hexagonal phase, as already described in our previous work [19], was also observed after the operation of BSCF membrane tubes in the demonstrator module of Kriegel et al. at positions below 850 °C [22]. The reversible separation of the original cubic BSCF into a Ba- and Co-enriched hexagonal and a Sr- and Fe-enriched cubic polymorph was reported by several research groups [41,42,43,44]. However, how the biphasic nature affects the strength of the BSCF is not known. In general, precipitations in the solid phase implicate micro residual stresses due to strain differences. During the transformation of the cubic to hexagonal BSCF polymorph, Svarcova calculated a volume decrease of 7–9% with a simultaneous increase in the non-stoichiometry δ [44]. To the authors’ understanding, the resulting micro residual stresses, as well as the newly formed incoherent interfaces at the grain boundaries, could also contribute to the strength degradation of the BSCF as a result of the module operation. Exactly at the fracture origin, with temperatures of about 750 °C, the decomposition of the cubic BSCF on the air side progresses most rapidly and should be mainly completed within the 1800 h operation [42].

Chromium and sulfur-rich areas in the EDS spectrum of the BSCF, as well as surface particles, were already observed in the hot zone along the outer surface in [19]. The latter, however, did not occur in the micrograph [19]. Sulfur contamination from grease or feed air might lead to the formation of Ba(Sr)SO_4_ crystals. Dark needles on the feed side down to a depth of 10 µm proved chromium poisoning, presumably by the formation of (Ba,Sr)CrO_4_. Due to the small depth of the microstructural change and its position on the outer surface, the influence on the strength is probably negligible and not measurable with the selected brittle ring method. 

Aluminum and silicon are present at the fracture position in the form of oxides and presumably originate from the insulation material, which was designated as highpurity Al_2_O_3_ fiber insulation. Remarkably, significantly less aluminum than silicon passes from the fiber material, with an Al:Si ratio of 31:1, to the outer surface of the membrane tube (see Table 3). On the membrane tube, the ratio was 1:5, indicating a predominant reaction of silicon with the membrane and not just loose particles of fiber insulation adhering to the membrane. The silicon contamination offered a possible explanation for the blue coloration. After permeation studies of LSCF and YSZ, Viitanen et al. [45] observed discoloration due to a SiO_2_ layer. Siloxane-containing grease in the valves was identified as the silicon source. After changing the grease, the discolorations no longer occurred. Schlehuber [46] attributed a similar blue discoloration after a 3000 h permeation through LSCF to a continuous Sr-Si oxide layer. The physical background of the blue coloration lies in the interference of differently refracted or diffracted light radiation at the thin silicon oxide layer. Since both barium and strontium contents clearly increased at the discolored fracture origin (in relation to the Co and Fe content), strontium-barium silicate glass could also have formed [47]. The presence of an amorphous Si compound also explains why no other phase besides BSCF was identified in the XRD experiments. While Viitanen et al. [45] were able to measure a reduction in the permeation rate as a result of the SiO_2_ layer formation, this was not observed by Schlehuber [46]. For the membrane tubes in the pilot membrane module, however, this is not superficially relevant since hardly any permeation occurs at the position of the fracture origin (T << 750 °C) anyway. Since no surface layer can be detected in the micrograph, the layer thickness of the Si-oxide must be below 1 µm and, thus, presumably has little influence on the strength. 

The observed strength decreases due to thermal shock (series A), and thermal cycling (series D) was unexpectedly high for the thin membrane segments. Approximate calculations resulted in a thermal shock parameter R_1_ of 150 K according to EN 820-3, which is better than the values determined for Al_2_O_3_ (54 K) and HIP-AlN (119 K) [48]. Similarly, as measured in the membrane module (Figure 2d), the rapid cooling of the ring specimens occurred simultaneously from the inside and outside. The specimens of series D, however, were separated out after the cycling of the membrane tubes in the laboratory module and could therefore build up higher stresses due to axial strain rest and temperature gradients between the feed and permeate sides. The cooling conditions in the laboratory module (720 °C after 10 min) were approximately the same as the cooling conditions in the A1 series of the thermal shock tests (700 °C after 10 min). While the residual strength was still 152 MPa (Δσ_0_ = −5%) after one cycle, where a drop to 96 MPa was determined after 20 cycles (Δσ_0_ = −40%). It can be assumed that smaller temperature amplitudes in the module can also have a similar cumulative effect on the strength of the membranes. Yáng et al. [49] observed several millimeters-long cracks in a BSCF dilatometer sample after 10 cycles between room temperature and 1000 °C in the air with very low heating rates of 60 K/h This was attributed to the repeated length change of ± 0.4% due to chemical expansion. In our specimens, few microcracks were identified after thermal shock. However, crack lengths and densities could not be correlated with the cooling rate and residual strength, respectively. The measured cooling rate in the membrane module −100 K/min was between series A1 (Δσ_0_ = −7%) and A2 (Δσ_0_ = −33%). Although thermocouples near the closed membrane end measured the temperatures during an emergency shutdown (Figure 2d), the measurement position and the interval of one minute caused great uncertainty. It is possible that the real cooling inside was faster, especially during the transition to the hot zone, as a result of the inflowing cold air.

### 4.3. Assessment of Membrane Failure under Static Axial Loads

Before defining the experimental layout for the static loading of membranes, we estimated the characteristic fracture stress for short-time loading to be at 850 °C via the size effect equation (7) using Weibull parameters determined at 850 °C in [38] and maximum tensile stresses of 26 kPa caused by the weight of a membrane. In a worst-case estimation, the effective volume of a membrane corresponded to the whole membrane volume of approx. 20,000 mm^3^. The calculated characteristic fracture stress of one membrane amounted to 32 MPa. This is the reason why we did not expect membrane fractures under a 30 to 60 kPa axial load even after several hundreds of hours. However, the difference between the prediction and experimental observation with fractures under 30 to 60 kPa in the axial load, thus, provides three orders of magnitude. The possible reasons for this discrepancy will be discussed in the following. 

In addition to the above-mentioned axial stresses, the membrane tubes were also subjected to the axial temperature gradient, aging, and slow heating. In contrast to the module operation, no oxygen partial pressure gradient was set, resulting in the absence of permeation and chemical expansion. The failure, thus, must be caused by a superposition of creep deformation and subcritical crack growth.

An estimation of the total creep strain using the secondary creep rate in the air (no temperature gradient assumed, T = 850 °C, σ = 30 kPa, d = 18 µm) resulted in $\dot{\varepsilon }$ = 2.3 × 10^−13^ s^−1^ [50] and the maximum time of 460 h until the creep rupture yielded a strain of 3.7 × 10^−7^. Related to the membrane length of 500 mm, only a 0.2 µm change in the length could thus be expected, which also explains why no strain was visible on the serial photographs. In the literature, initial damage due to grain boundary widening was only to be expected from local strains of 0.2%, and the macroscopic creep rupture was observed at a 40% total strain [50]. Transferred to our BSCF membranes, grain boundary widening should not occur before 280 years.

Estimates can also be made for the failure due to subcritical crack growths with Equation (8). With times of t_1_ = 10 s and t_2_ = 460 h, a normalization stress of σ_0V,1_ = 121 MPa from the short time fracture tests at 850 °C [38] and the subcritical crack growth parameter *n* = 40 [51], the normalized stress at time t_2_ amounts to σ_0V,2_ = 89 MPa, giving a single membrane tube a failure probability of 63% at 23 MPa. However, such high loads are not present in the static test. Only the assumption of an extremely small subcritical crack growth parameter of *n* = 1.84 could result in σ_0V,2_ = 0.18 MPa, and a 63% fracture probability of an axially loaded membrane with 46 kPa. A very low parameter *n* = 12 has been reported in the literature for the OTM membrane GCO [51], while more typical values lie above *n* = 20. However, subcritical crack growth is usually analyzed at room temperature. A high influence of the test temperature was proven by Choi et al. for Al_2_O_3_ with 96% purity with a decrease from *n* = 50 at room temperature to *n* = 7 at 1000 °C [52]. Nevertheless, the value *n* = 1.84 appears to be clearly too low.

One reason for the failure of the membrane tubes in the static test could, therefore, also be unknown stresses, such as resonance with exciting aggregates in the environment. Some analyses of the natural frequencies and shapes of the membrane tubes, as well as frequency measurements, are described in Appendix E. Overall, a vulnerable region with nine closely spaced natural modes was identified between 7 and 8 kHz. Numerous interferences of exciting aggregates were transmitted by ground vibrations. In new module designs, the stiff bolting of the pressure vessel in the floor should be replaced by better-damped systems. In future tests, it should also be analyzed whether excitation in the endangered area can actually trigger a failure.

### 4.4. Predicted Failure Probability Versus Experimentally Observed Fracture

The FE simulation of the stress distribution showed very high stress and fracture probabilities directly after temperature and pressures were applied (step 1). The chemically induced stresses are responsible for this due to volume expansion on the permeate side. These strain differences between the inner and outer surface of the membrane occurred at a radial distance of 920 µm. The thermal gradient, on the other hand, occurred in the axial direction along a distance of 15–20 cm, with almost no strain constraints in this direction. The high stresses of up to 70 MPa on the outside of the membrane were not implausible. The results are in qualitative agreement with simulations by Kwok et al. [24]. The authors calculated maximum tangential and axial stresses of 20 MPa for the tubular-supported BSCF membranes where the outer dense functional layer was 50 µm. The larger wall thickness of our membranes led to higher stresses on the outside due to higher strain constraints. Euser modeled tangential stresses of 120 MPa for 200 µm thick LSCF membranes using a 1D finite volume mesh in the radial direction and isothermal conditions [26]. Kriegel et al. pointed out that chemical strains are relevant to failure and cannot be compensated by external compressive forces. According to unspecified calculations in COMSOL, the maximum tensile stresses for the tubular or capillary membranes under similar pressure-temperature conditions are 28 MPa [53]. 

As a result of the creep deformation in the simulation of step 2, axial and tangential stresses relax in the compressive stress range on the inside and in the tensile stress range on the outside. The radial stresses remain relatively unchanged after 1000 h and partly form the maximum stress component on the outside. However, the local maximum stress of 43 MPa at the position x = 0.21 was noticeable after 1000 h of creep. This local maximum does not perfectly coincide with the position of the fracture origin at 0.25 ± 0.02 (12.5 ± 1 cm). However, the simulation results are based on the axial temperature gradient from Figure 2c, which was measured only one time by manually extracting the data of the thermocouple step by step. However, the local creep rates and the chemical strain are based on the error-prone determined temperature gradient. While the chemical strain initially sets in progressively above 500 °C (compare [54]), a measurable creep relaxation that exponentially depends on temperature starts later. The local maximum stress occurred after 1000 h of creep time since the chemically induced stresses can only relax effectively at sufficiently high temperatures of >750 °C. Furthermore, it is quite conceivable that the input parameters of the model, which are derived from error-prone temperature measurements and extrapolated creep parameters from the literature (see Appendix D), are not precise enough to accurately predict the position of the maximum stress. Kwok et al. simulated asymmetric BSCFZ membranes considering the chemical strain and an axial temperature gradient, but the creep rate was independent of the oxygen stoichiometry [30]. No local stress maximum appeared in their study. However, the results are comparable to our own work in the general trend of stress relaxation and the reduction in failure probability. To our best knowledge, further reference to the literature on the simulation of creep relaxation after exposure to the oxygen partial pressure gradient does not exist.

The simulated emergency shutdown in step 3 and the resulting pressure equalization has a significant effect on the reduction in the chemical strain contribution. The stress distribution in step 1 is inverted but no longer reaches 70 MPa due to the stress relaxation that occurred previously. The simulation of cooling was performed as a transient simulation to identify the temperature gradients due to “hot spots” inside the membrane wall. However, this assumption was not confirmed. The overall emergency shutdown appears to be less critical than previously suspected based on the clustered failures. However, the exact local time-temperature-pressure histories during the emergency shutdown are unknown and are only an estimate. Indications that the change in partial pressures and the resulting surface exchange and transport processes may also be critical are given by Zolochevsky et al. [25]. In addition to the known maximum of the first principal stress on the oxygen-rich side of the membrane, they identified a maximum Mises stress that occurred after 27 s on the oxygen-deficient side based on an isothermal transient FEM simulation in Ansys. This can exceed the compressive strength.

When calculating fracture probabilities, the test method for determining the Weibull parameters and selecting the failure hypothesis can also significantly influence the result. By recognizing the magnitude of the effective volume, the combination of the HT-scaled Weibull parameters with the PIA hypothesis represents the most pessimistic case, whereas optimistic assumptions are present when the Weibull parameters from the ball-on-three-balls test are combined with the NS theory. Regardless of the assumptions made, the calculated probability of fracture is 100% after the application of pressure and temperature. After 1000 h of stress relaxation, a fracture probability between 7 and 56% is predicted, and after emergency shutdown, a fracture probability of 85–100%. This does not agree with experimental observations since long-term test 1 membrane failures, only in isolated cases, had already occurred during/immediately after heating [19]. Most of the membranes failed during or after the emergency shutdown. 

Reasons for the deviation between the simulation (fracture) and experimental observation (no fracture) directly after setting the permeation conditions (time t_1_) are numerous. We simulated the simultaneous application of pressure and temperature profiles, whereas in the module operation, the pressures were applied before heating. This allowed creep relaxation to occur during the long heating process (with a heating rate of 300 K/h) before the maximum temperature was reached. Furthermore, a linear course of the non-stoichiometry δ was assumed in a simplified manner. According to [23], however, the curve δ versus thickness was more asymptotic, which presumably resulted in a smaller effective volume. Resultant higher stresses on the outside can be relieved more quickly by creep.

**Figure 15:**
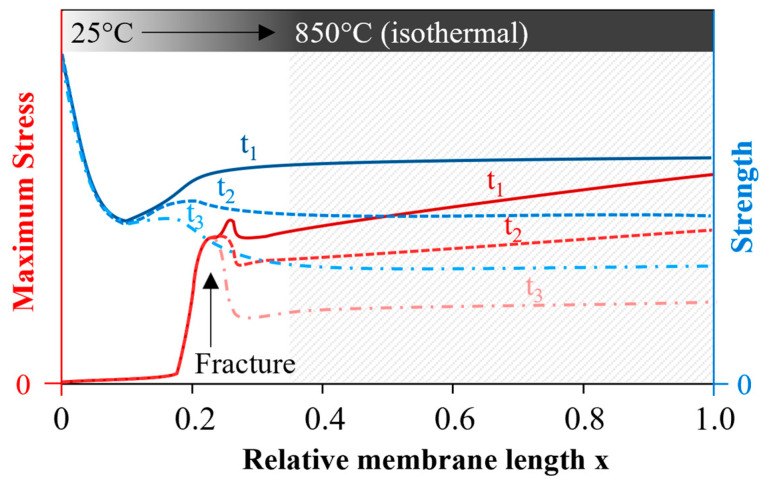
Possible progression of maximum stress and strength for times t_1_ < t_2_ < t_3_; fracture occurred at time t_3_ since max. stress > strength.

A possible scenario of why the membranes in the module failed, not immediately but after some permeation time, at the blue discolored areas is sketched in Figure 15. Even though the exact position and temporal formation of the local stress maximum are certainly different in reality, its existence is a plausible finding. In addition, it has been demonstrated that the strength decreased with exposure time and exhibited a characteristic temperature dependence with an intermediate temperature minimum [38,55]. Therefore, Figure 15 plots the maximum stresses (red) and strength (blue) as a function of the membrane length for different times t_1_ < t_2_ < t_3_. At time t_3_, a blue curve at the position of the local stress maximum exceeded the red curve, and the rupture occurred. It is possible that already at time t_2,_ additional unconsidered stresses were superpositioned by the emergency shutdown, which triggered a fracture. To be mentioned here are the forces of the compressed air flowing out of the vessel, the vibrations, and the real surface temperatures, which could deviate considerably from the temperature measurement with the acquisition rate of only one measuring point per minute in Figure 2d. Appendix E gives further indications of a stronger strength degradation than assumed. Following the outlined hypothesis, the spontaneous membrane failure at time t_3_ without the influence of additional forces did not occur at all.

## 5. Conclusions

The industrial application of ceramic membranes for oxygen removal from the air requires gas-tight joined membrane components that guarantee a reliable permeation operation under oxygen partial pressure gradients at 850 °C. However, tubular BSCF membranes, when cooled at the open end, failed at typical positions as a result of the unintended changes to the operating conditions in the Oxycoal-AC membrane module [19].

In this work, the strength and strength degradation of the membrane material under near-application operating conditions were experimentally investigated and correlated with observed surface as well as microstructural changes. The main findings are:

- During the long-term tests in the module, coexisting hexagonal phase formed in the bulk BSCF, which can lead to micro residual stresses. A generally higher and more homogeneous temperature inside the module is therefore recommended.
- Near-surface secondary phases and overlying particles could be Ba(Sr)-chromates and -sulfates. The blue iridescent discolored surface at the typical fracture origin indicates the layer formation of amorphous (Sr,Ba)-silicate with silicon from the fiber insulation.
- The brittle ring tests performed showed a 15% reduction in strength degradation. This was attributed to aging with grain and pore coarsening.
- Using C-ring tests instead of brittle-ring tests, strength at the glazed surface can be investigated in future work. In addition, the question of how the strength of BSCF changes under permeate-side conditions should be investigated.
- The strength decrease due to thermal shock and thermal cycling with low temperature gradients indicates subcritical crack growth. Microcracks could be identified in isolated cases, but not quantitatively related to the residual strength. The residual strength reduced from 5 to 40 % between the 1st and 20th cycle.
- Long-term tests of membranes under low axial tensile stress in air showed an unexpectedly high susceptibility to subcritical crack growth or creep fractures and should be further investigated.

Results from the transient simulation of the stress distribution and fracture probability calculation are summarized as follows:

- In the finite element model, high tensile stresses of 70 MPa were obtained on the outside of the membrane, which were largely due to the chemical strains. According to the simulation results, failure should occur immediately after the pressure is applied to the closed end of the membrane.
- A scenario was derived to explain the membrane ruptures during emergency shutdown or after long periods. According to this scenario stresses formed by chemical expansion at temperatures above 500 °C can effectively relax during permeation not below temperatures of 750 °C. The position of the local stress maximum matches with the experimentally observed fracture position. Failure is initiated as the time, temperature and thus position dependent strength falls below the local stress maximum.

## 6. Outlook

Some important recommendations can be derived for the technology of oxygen separation using tubular OTM membranes. By replacing the adhesive seal with a high-temperature-resistant braze, the axial temperature gradient can be shifted to the metallic sleeve. Recently, major advances have been presented to minimize the strength degradation of reactive brazed BSCF-AISI314 joints during isothermal aging [56]. This implies two major advantages. First, the entire membrane area can then contribute to permeation. Second, the entire membrane tube will be under chemically induced stresses that will relax due to creep. Although the chemical strains of BSCF are a requirement for oxygen permeation, the induced stresses can be additionally reduced by decreasing the wall thickness of the membrane tubes and the resulting strain restraint. This requires more homogeneous wall thicknesses than cold isostatic pressing can deliver. However, the technology for the one-sided closure of extruded tubes has been developed very far in the past decade. Recently, 250 h permeation operations have also been demonstrated with asymmetric tubular membranes [20]. There are now promising doping concepts for the material BSCF, which have a higher chemical resistance strength but also thermal shock resistance [57]. With regard to process control, however, rapid changes in processing conditions should be avoided even during an emergency shutdown. This can be solved constructively by using throttle valves on the permeate side to prevent the inflow of cold air. 

## Figures and Tables

**Table 1:** Overview of tested brittle ring series.

	Edge Condition	Wall Thickness Inhomogeneity	Pre-Treatment	Number of Specimens
Reference 1	Polished	Measured for each specimen	-	22
Reference 2	Ground	Not measured	-	23
Series A	Polished	<150 µm	Thermal shock	4 x ~20
Series B	Polished	<150 µm	1000–1800 h aged in module operation	25
Series C	Ground	Not measured	1000–1800 h aged in module operation, Blue colorized zone	19
Series D	Polished	Measured for each specimen	Thermal cycling	8

**Table 2:** Steps and corresponding parameters in the FE-simulation.

Step (No.)	Type	Duration	Constraints	Temperature	Load
Initial (0)	s	-	Fixed, symmetry	-	-
Start (1)	s	-	As in step 0	Axial gradient	1 MPa pressure (o), 0.095 MPa tensile (i)
Permeation (2)	t	1000 h	As in step 1	CREEP subroutine	
Shut down (3)	t	120 s	As in step 2	Reduction by 10% (i & o)	Linear decrease to 0 MPa (i and o)

i = inside, o = outside, s = static, t = transient.

**Table 3:** Mean values and standard deviation of EDS and WDS analysis in mol%.

Analysis Method: Position *	O	Al	Si	S	Cr	Fe	Co	Sr	Ba
WDS: sintered BSCF ^14^	56.4 ± 0.1	-	-	-	-	4.3 ± 0.1	17.0 ± 0.1	11.3 ± 0.1	11.0 ± 0.1
EDS: fracture position, inner surface ^4^	±0.4	-	-	-	-	4.2 ± 0.1	16.9 ± 0.5	10.7 ± 0.2	11.3 ± 0.1
EDS: fracture position, outer surface ^4^	55.5 ± 0.5	1.4 ± 0.4	6.9 ± 0.1	0.5 ± 0.3	1.9 ± 1.1	1.8 ± 0.5	7.1 ± 1.3	10.4 ± 1.0	14.4 ± 1.2
EDS: near closed membrane end, outer surface ^3^	59.1 ± 0.4	-	-	3.8 ± 0.4	-	3.8 ± 0.1	12.8 ± 0.5	9.7 ± 0.2	10.8 ± 0.1
EDS: fiber insulation ^1^	67.6	31.4	1	-	-	-	-	-	-

* superscript marks the number of measuring points.

**Table 4:** Weibull parameters, effective volume, and resulting normalized strength of brittle ring tests of the reference series. * Scaled from room temperature to 850 °C.

	Edge Quality	Valid at	m [-]	σ_0_ [MPa]	V_eff_ [mm^3^]	σ_0V_ [MPa]
Reference 1	Polished	room temperature	6.2	162	6.2	218
Reference 2	Ground	room temperature	5.2	117	7.6	173
Reference 1 *	Polished	850 °C *	5.4 *	-	-	132 *

**Table 5:** Weibull parameters, effective volume, and resulting normalized strength of pre-treated brittle ring tests.

	Edge Quality	Pre-Treatment	m [-]	σ_0_ [MPa]	V_eff_ [mm^3^]	σ_0V_ [Mpa]
Series A1	polished	Thermal shock	6.4	151	6.0	200
Series A2			6.2	109	6.2	146
Series A3			1.9 ^2^	84 ^2^	29.4 *	497 *
Series A4			4.9 ^3^	16 ^3^	8.2	25
Series B	Polished	Aging	6.8	142	5.7	183
Series C	Ground	Chemical reaction	5.9	98	6.6	135
Series D	Polished	Thermal cycling	3.1	96	14.7 *	229 *

The number of failures due to the pre-treatment is indicated by superscript numbers; * cannot be applied technologically.

**Table 6:** Weibull parameters, effective volume and the resulting normalized strength of pre-treated brittle ring tests.

	Assumption	Brittle Ring Test			Ball-on-Three-Balls Test		
	Reference	Reference Series 1 *, See Table 4 (HT-Scaled to 850 °C)			[38]		
	m [-]	5.4 *			7.4		
	σ_0V_ [MPa]	132 *			121		
	Step	1	2	3	1	2	3
	σ_I,max_ [MPa]	70	43	47	70	43	47
NS	V_eff_ [mm^3^]	1993	277	2474	1804	131	1981
	P_f_ [-]	1	0.48	1	1	0.07	0.85
PIA	V_eff_ [mm^3^]	3312	354	4763	2837	166	3758
	P_f_ [-]	1	0.56	1	1	0.08	0.97

The number of failures due to the pre-treatment is indicated by superscript numbers; * cannot be applied technologically.

**Figure A1:**
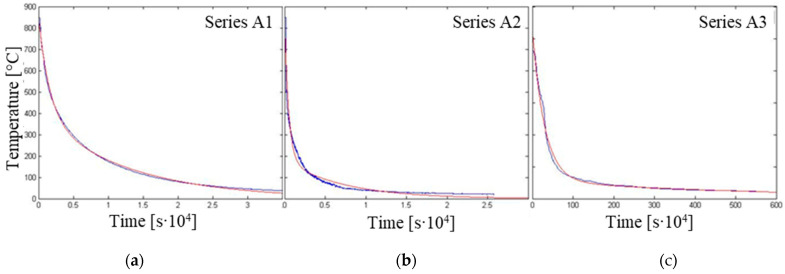
Measured (blue) and fitted (red) cooling profiles of thermal shock treatments (**a**) Series A1, (**b**) Series A2 and, (**c**) Series A3.

**Figure A2:**
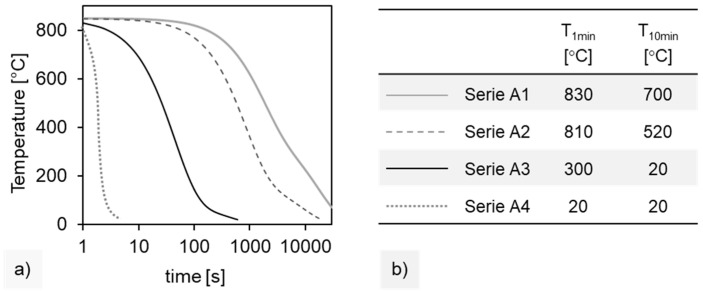
Comparison of fitted temperature-time profiles during the four thermal shock treatments (**a**) graphically, (**b**) by characteristic temperatures after 1- and 10-min cooling.

**Figure A3:**
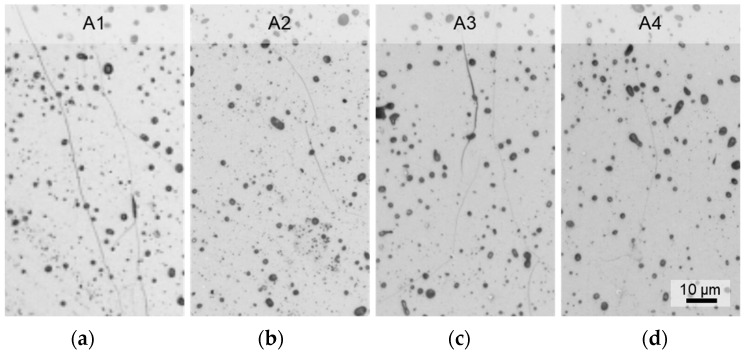
Exemplary light microscope images of polished cross sections of (**a**) series A1, (**b**) series A2, (**c**) series A3, (**d**) series A4 showing microcracks formed by subcritical crack growth.

**Figure A4:**
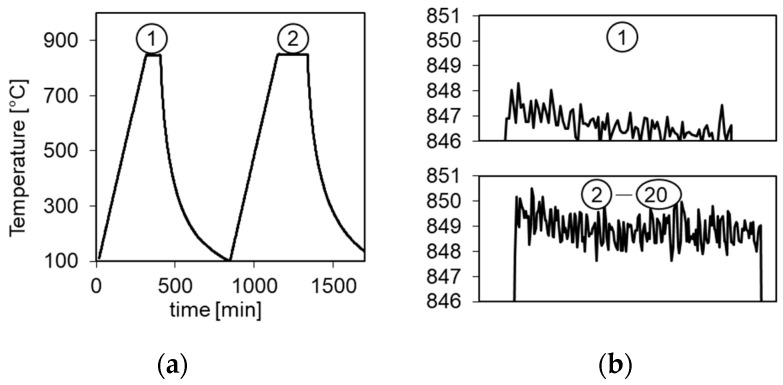
Measured temperature-time profile during thermal cycling treatment of tubular membranes. The temperature oscillation during the holding time was around ± 1 K. After adjusting the control parameters during the first cycle (**a**), a mean isothermal temperature of 849 °C was obtained in the following cycles (**b**).

**Figure A5:**
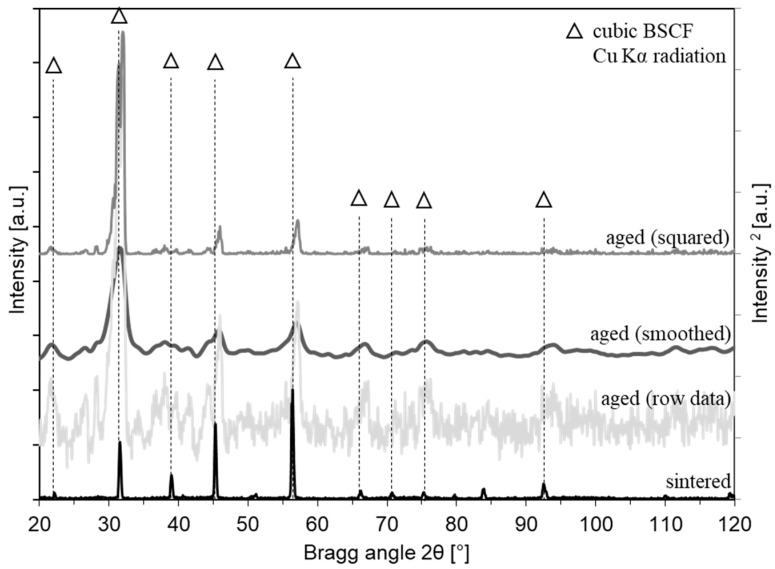
Cubic phase of sintered BSCF using X-ray diffraction with Cu Ka radiation and difficulties in phase analysis of the aged BSCF from the blue discolored area.

**Figure A6:**
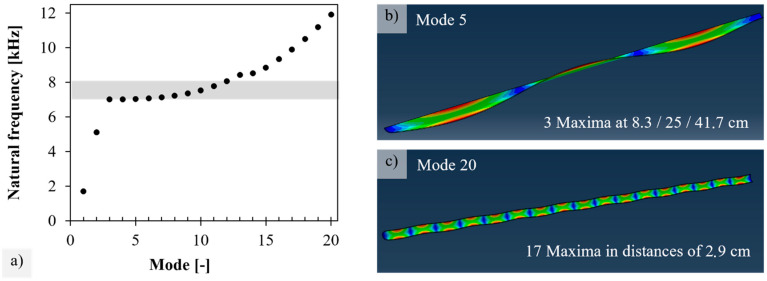
(**a**) Determined eigenfrequencies of the first 20 modes as well as exemplary eigenmodes at mode 5 (**b**) and 6 (**c**). The scaling factor of the displacements amounts to 10.

**Figure A7:**
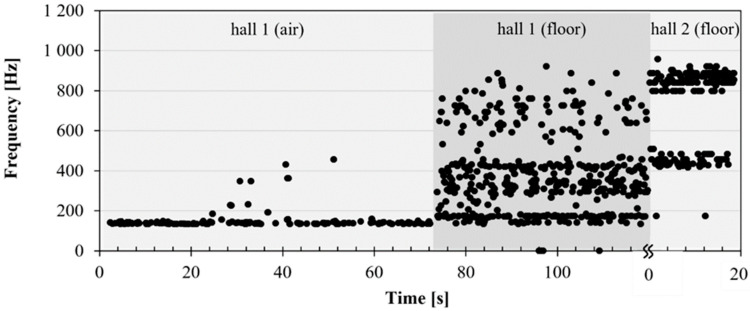
Frequency measurement in test hall 1 and test hall 2 outside module operation using Phyfox [58].

## Data Availability

The data presented in this study are available in the article or the appendices. The list of obtained fracture forces and will be available soon in RWTH Publications at [10.18154/RWTH-2022-06816]. The Fortran-code for the simulation subroutine in this study is available at https://zenodo.org/record/7071049#.Yx8Ght9CSUk.

## Appendix A

Three cooling curves were obtained from the logged temperatures of the three thermocouples and fitted to two weighted exponential functions using the least squares method of Levenberg—Marquardt optimization:

(A1) $$T=p_{1}\cdot e^{p_{2}\cdot t}+p_{3}\cdot e^{p_{4}\cdot t}$$

A comparison of measured (blue) and fitted (red) cooling curves are given in Figure A1.

The average value of the three fitting parameters a, b, c, and d is given in the following table for each series. Note, that the temperature could not be measured during water quenching in Series A4. The samples were removed from the water bath after 10 s and had assumed water temperature in the meantime.

**Table A1:** Fitting Parameters describing the cooling profile during thermal shock treatment.

	a [°C]	b [1/s]	c [°C]	d [1/s]
Series A1	449.7	−6.08∙10^6^	400.1	−7.73∙10^5^
Series A2	660.2	−1.233∙10^3^	188.5	−1.147∙10^4^
Series A3	255.2	−2.283∙10^2^	75.46	−2.395∙10^3^
Series A4	not determined			

With these fitting parameters the characteristic cooling profiles (a) and temperatures after 1 and 10 min of cooling (b) in Figure A2 are obtained.

Exemplary optical microscope images of the polished cross-sections of Series from A1 to A4 are given in Figure A3.

## Appendix D

**Table A2:** Input parameters for the simulation of a tubular BSCF membrane during three steps: (1) start of permeation, (2) stable permeation, (3) emergency shut down. The Fortran-Code of the Subroutine is available in S1.

T [°C]	CTE α_total_ [10^−6^ K^−1^]			Young’s Modulus ^(3)^ [GPa]	Themal Conductivity [W/mK]	Specific Heat Capacity [J/kgK]
	Outer ^(1)^	Inner ^(2)^				
20	14.70	14.70		63.2	0.984	460.0
100	14.77	14.77		52.7	0.961	512.9
200	13.84	13.84		46.8	0.985	574.3
300	12.85	12.85		44.5	-	-
400	13.25	13.25		51.3	-	-
500	14.10	16.00		52.9	1.294	614.4
600	15.81	17.77		53.3	-	-
700	17.69	19.53		50.5	-	-
800	18.22	20.57		48.6	2.265	680.8
900	18.86	21.62		47.0	3.095 ^(4)^	706.3 ^(4)^
Explanations: ^(1)^ CTE (coefficient of thermal expansion) from own dilatometry in the air; ^(2)^ CTE plus CCE (coefficient of chemical expansion) at the permeation side above 500 °C [52]; ^(3)^ Assumption of similar Young’s moduli for feed and permeate sides. Slightly higher values of the Young’s modulus were measured at 10^−5^ mbar. At the present permeate pressures of 5∙10^−2^ bar, these were no longer significant; ^(4)^ Measured at 960 °C; ^(5)^ Constants: Poisson ratio 0.25, density 5.28 g/cm^3^; ^(6)^ The exponential cooling by 10% for 0 < t < 120 s analogous to Figure 2d is represented by the following temperature curve: (A2) $\frac{T_{\left(t\right)}}{T_{\left(t=0\right)}}=-0.01727\cdot \ln\left(t+\frac{1}{e}\right)+0.98273$ The surface temperatures were thus specified as position-dependent, and the temperatures within the membrane resulted from the thermal conductivity and heat capacity in the thermomechanical model. ^(7)^ The steady-state creep rate was calculated using the parameters in the table below from reference [49]. Creep rates were determined in this reference for T > 850 °C, but were also assumed for T < 850 °C. The creep rate was higher in the vacuum than under the air and was therefore linearly interpolated between the inner and outer surfaces via the oxygen partial pressure (p_O2, outside_ = 0.021 MPa and p_O2, inside_ = 0.005 MPa): (A3) $\dot{\varepsilon }=A{\left(\frac{1}{d}\right)}^{p}{\left(pO_{2}\right)}^{m}\sigma^{n}\exp\left(-\frac{E_{a}}{RT}\right)$ where the corresponding constants are listed in the following table.						
average Ea	average A	n	m	p	R	d
mJ/mol	mm^1.7^/(MPa^1.56^·s)	-	-	-	mJ/(molK)	mm
3.38 × 10^8^	290	1.7	−0.14	1.7	8314	0.018

## Appendix E

In the membrane module, no attention was paid to possible resonances when designing the components. The membranes were inserted via tight fits and rubber-ring sealings into the water-cooled flange, which was bolted to the lower vessel. The lower vessel was rigidly bolted to the flange and the concrete hall floor. This might lead to a weakly damped system in which resonance could occur due to excitation near the natural frequency of the membranes. Conceivable excitation aggregates are the compressor, the vacuum pump, or high-frequency pulsators for fatigue testing.

To identify the natural frequencies and mode shapes of the membranes, a modal analysis was performed using the same model as in Figure 4b in the initial condition. The intent of the study was to evaluate the probability of membrane failure due to resonance with a characteristic frequency of aggregates in the environment.

The natural frequencies of the first 20 modes are shown in Figure A6a. The first natural frequency was 1.7 kHz. A total of nine modes occurred between 7 and 8 kHz. This range is particularly susceptible to resonance. In case of excitation close to a certain natural frequency, the membrane tube would assume the corresponding eigenmode with an equidistant node and wave positions. Figure A6b and c show an example of the eigenmodes 5 and 20. The number of nodes increases with the mode number, but the amplitude decreases. There are several modes with maxima at the typical fracture position of x = 0.25.

The rotating units in the vicinity have operating frequencies that are clearly too high or too low to be considered direct exciters. The compressor operates at 88.2 kHz, the vacuum pump at 90 or 108 kHz, and the vibration testing by means of various testing machines in the neighboring test hall 1, which was performed at 10–150 Hz. The frequency measurement, as shown in Figure A7, revealed numerous harmonics and interferences, which were measurable, especially on the floor. In test hall 2, no measurement was possible during or after the module operation, running the compressor, and vacuum pump. The measured floor vibrations originated from the test rigs for fatigue strength investigation, which were below the first natural frequency, resulting in why they could not cause any resonance of the membrane tubes. No vibrations were obviously transmitted from hall 1 to hall 2 via the floor.
